# Marijuana use and DNA methylation-based biological age in young adults

**DOI:** 10.1186/s13148-022-01359-8

**Published:** 2022-10-26

**Authors:** Drew R. Nannini, Yinan Zheng, Brian T. Joyce, Tao Gao, Lei Liu, David R. Jacobs, Pamela Schreiner, Chunyu Liu, Steve Horvath, Ake T. Lu, Kristine Yaffe, Stephen Sidney, Philip Greenland, Donald M. Lloyd-Jones, Lifang Hou

**Affiliations:** 1 Department of Preventive Medicine, Northwestern University Feinberg School of Medicine, 680 N. Lake Shore Drive, Suite 1400, Chicago, IL 60611 USA; 2 Division of Biostatistics, Washington University, St. Louis, MO USA; 3 Division of Epidemiology and Community Health, School of Public Health, University of Minnesota, Minneapolis, MN USA; 4 Department of Biostatistics, Boston University School of Public Health, Boston, MA USA; 5 Department of Human Genetics, David Geffen School of Medicine, University of California Los Angeles, Los Angeles, CA USA; 6 Department of Biostatistics, Fielding School of Public Health, University of California Los Angeles, Los Angeles, CA USA; 7 University of California at San Francisco School of Medicine, San Francisco, CA USA; 8 Kaiser Permanente Division of Research, Oakland, CA USA

**Keywords:** Marijuana, Epigenetic age acceleration, Alcohol, CARDIA, Aging

## Abstract

**Background:**

Marijuana is the third most commonly used drug in the USA and efforts to legalize it for medical and recreational use are growing. Despite the increase in use, marijuana’s effect on aging remains understudied and understanding the effects of marijuana on molecular aging may provide novel insights into the role of marijuana in the aging process. We therefore sought to investigate the association between cumulative and recent use of marijuana with epigenetic age acceleration (EAA) as estimated from blood DNA methylation.

**Results:**

A random subset of participants from The Coronary Artery Risk Development in Young Adults (CARDIA) Study with available whole blood at examination years (Y) 15 and Y20 underwent epigenomic profiling. Four EAA estimates (intrinsic epigenetic age acceleration, extrinsic epigenetic age acceleration, PhenoAge acceleration, and GrimAge acceleration) were calculated from DNA methylation levels measured at Y15 and Y20. Ever use and cumulative marijuana-years were calculated from the baseline visit to Y15 and Y20, and recent marijuana use (both any and number of days of use in the last 30 days) were calculated at Y15 and Y20. Ever use of marijuana and each additional marijuana-year were associated with a 6-month (*P* < 0.001) and a 2.5-month (*P* < 0.001) higher average in GrimAge acceleration (GAA) using generalized estimating equations, respectively. Recent use and each additional day of recent use were associated with a 20-month (*P* < 0.001) and a 1-month (*P* < 0.001) higher GAA, respectively. A statistical interaction between marijuana-years and alcohol consumption on GAA was observed (*P* = 0.011), with nondrinkers exhibiting a higher GAA (*β* = 0.21 [95% CI 0.05, 0.36], *P* = 0.008) compared to heavy drinkers (*β* = 0.05 [95% CI − 0.09, 0.18], *P* = 0.500) per each additional marijuana-year. No associations were observed for the remaining EAA estimates.

**Conclusions:**

These findings suggest cumulative and recent marijuana use are associated with age-related epigenetic changes that are related to lifespan. These observed associations may be modified by alcohol consumption. Given the increase in use and legalization, these findings provide novel insight on the effect of marijuana use on the aging process as captured through blood DNA methylation.

**Supplementary Information:**

The online version contains supplementary material available at 10.1186/s13148-022-01359-8.

## Background

Marijuana is the third most commonly used drug after alcohol and tobacco, with approximately half of US adults having ever used marijuana and 10% having used marijuana in the past month [1]. Marijuana has been subject to ongoing legal and social debates, including its use for medical therapies and recreational use. As a medical therapy, marijuana is used to reduce chemotherapy-induced nausea and vomiting [2] and chronic neuropathic pain [3], although it increases risk of cardiovascular disease [4–7], respiratory illness [8, 9] and metabolic disorders [10]. Marijuana use has also increased over the past several decades, coincident with laws and regulations [11]. Due to the increase in use and increasing number of states legalizing recreational marijuana, studies are needed to evaluate its health effects, in particular its cumulative effects on health. While previous studies observed associations between marijuana and age-related health outcomes, the effect of marijuana on the aging process at a molecular level has not received sufficient attention.

Several molecular markers have been proposed to quantify biological age, including epigenetic age as estimated from age-related DNA methylation biomarkers [12, 13]. Moreover, the discrepancy between chronological age and epigenetic age is used to calculate epigenetic age acceleration (EAA), where a higher value represents an older epigenetic age relative to one’s chronological age and vice versa. Several epigenetic age and EAA metrics have been developed, including those by Horvath, Hannum, Levine, and Lu, and have been associated with multiple age-related outcomes, such as disease, physical functionality, and mortality [13–16].

Lifestyle factors, such as alcohol consumption, tobacco smoking, physical activity, and diet, have been shown to accelerate or decelerate epigenetic aging relative to chronological age [17–19]. Cumulative and recent exposures were also shown to have varying associations with EAA. For example, cumulative alcohol consumption was positively associated with EAA [20], whereas recent consumption exhibited inverse associations [17], suggesting possible difference in effects of cumulative and recent exposures on EAA. However, studies examining the effect of marijuana, both cumulative and recent use, on epigenetic aging remain limited. Given the limited data on marijuana age-related epigenetic changes, we investigated the association between marijuana and EAA in the Coronary Artery Risk Development in Young Adults (CARDIA) Study, in which marijuana has been longitudinally collected.

## Methods

### Study sample

Details of the CARDIA study design, recruitment, and examinations have previously been documented [21]. Briefly, CARDIA was designed as a population-based cohort study investigating the determinants and development of subclinical and clinical cardiovascular disease. From 1985 to 1986, 5115 Black and White study participants ages 18 to 30 years were recruited from four centers across the US and received in person examinations at baseline (year 0 [Y0]), and at Y2, Y5, Y7, Y10, Y15, Y20, Y25, and Y30.

### Marijuana use measurements

Marijuana use was obtained at baseline and at each follow-up examination by asking participants “Have you ever used marijuana?”, “About how many times in your lifetime have you used marijuana?”, and “During the last 30 days, on how many days did you use marijuana?” We considered four variables to capture cumulative and recent use of marijuana at Y15 and Y20. Two binary marijuana variables indicated if a participant has ever used marijuana (cumulative use) and used in the last 30 days (recent use). A continuous variable quantified the number of days of marijuana use in the last 30 days (recent use). We also estimated a continuous variable capturing cumulative marijuana use, i.e. ‘marijuana-years’, as previously described [22, 23]. Briefly, we assumed marijuana use in the last 30 days reflected use during the time period between examinations, where a marijuana-year is equivalent to 365 days of marijuana use. We then estimated cumulative marijuana-years by summing the total number of days of marijuana use from baseline to Y15 and Y20 separately and dividing by 365.

### DNA methylation profiling

Methylation profiling and DNA quality control have been described elsewhere [24–26]. Briefly, a subset of 1200 randomly selected participants with available whole blood repeatedly collected at both Y15 and Y20 (2400 total samples) underwent DNA methylation profiling using the Illumina MethylationEPIC Beadchip. Data preprocessing and quality control were performed using the R package ENmix [27] using default parameter settings. Methylation measurements with a detection *P* < 1E−06 or less than 3 beads were defined as low quality. A total of 6209 CpG sites with a detection rate < 95% and 87 samples with low-quality methylation measurements > 5% or extremely low intensity of bisulfite conversion probes (less than 3 × standard deviation of the intensity across samples below the mean intensity) were excluded from further analysis. An additional 95 samples were defined as extreme outliers via the average total intensity value [intensity of the unmethylated signal (*U*) + intensity of the methylated signal (*M*)] or *β* value [*M*/(*U* + *M* + 100)] across all CpG probes and Tukey’s method [28]. A model-based background correction method was applied to samples using ENmix and correction for dye bias was performed using RELIC [29]. Quantile-normalization of *M* or *U* intensities for Infinium I or II probes were performed separately, respectively. Low-quality methylation values and *β* value outliers (via Tukey’s method) were set to missing. After data processing, the final methylation dataset for epigenetic age calculation contained 1042 and 957 samples at Y15 and Y20, respectively.

### Epigenetic age calculation

We calculated four epigenetic age estimates. Horvath’s age, intrinsic epigenetic age acceleration (IEAA), was estimated using 353 CpGs and is associated with cell-intrinsic aging [13]. Hannum’s age, extrinsic epigenetic age acceleration (EEAA), was estimated from 71 CpGs and is associated with immune system aging [14]. Levine’s age, PhenoAge acceleration (PAA), was estimated using 513 CpGs and is associated with physical functionality and comorbidities [15]. Lastly, Lu’s age, GrimAge acceleration (GAA), was estimated from 1,030 CpGs and is associated with lifespan [16]. The DNA-methylation epigenetic age estimates were calculated using the publicly available online calculator (https://dnamage.genetics.ucla.edu/new). EAA was calculated from the residuals from a linear regression model for each epigenetic age regressed on chronological age.

### Statistical analysis

We conducted statistical analyses to examine the associations between each EAA estimate (outcome variables) and the cumulative and recent marijuana use variables (independent variables) collected at Y15 and Y20. Multiple linear regression and quantile regression were performed to evaluate the associations between EAA and the marijuana variables and generalized estimating equations (GEE) were evaluated to examine these associations across time. Interaction and stratified analyses were performed to investigate the joint association of marijuana use with alcohol consumption, tobacco smoking status, race, and sex on GAA during Y15, Y20, and GEE analyses. Alcohol consumption was classified according to CDC guidelines, i.e. nondrinkers (*n*_Y15_ = 429, *n*_Y20_ = 387), light drinkers (≤ 3 drinks per week; *n*_Y15_ = 204, *n*_Y20_ = 142), moderate drinkers (4–7 drinks for females and ≤ 14 for males per week; *n*_Y15_ = 241, *n*_Y20_ = 231), and heavy drinkers (> 7 drinks for females and > 14 drinks for males per week; *n*_Y15_ = 149, *n*_Y20_ = 123) during stratified analysis [30]. Models were adjusted for sex, race, center, education, tobacco smoking status, cumulative packs of cigarettes, body mass index, physical activity, and alcohol consumption. Associations were declared significant if *P* ≤ 0.05. All statistical analyses were performed using SAS 9.4.

## Results

### Sample characteristics

Characteristics of participants who underwent DNA methylation profiling at Y15 and Y20 have been described previously and were not found to be different from participants who did not undergo methylation profiling in the CARDIA cohort [24]. Table 1 presents the summary characteristics for study participants who underwent methylation profiling at Y15 and Y20 by marijuana-year. In total, 1023 and 883 participants had available methylation and marijuana data at Y15 and Y20, respectively. At Y15 and Y20, 71.9% and 70.1% of participants reported that they have used marijuana and 13.7% and 12.8% used marijuana in the last 30 days, respectively. At both examination years, participants with at least 1 marijuana-year exhibited higher EEAA, PAA, and GAA compared to participants who never used marijuana.

**Table 1:** Descriptive statistics of the study sample at examination years 15 and 20

	Year 15			Year 20		
	0 MJ Years	< 1 MJ Years	≥ 1 MJ Years	0 MJ Years	< 1 MJ Years	≥ 1 MJ Years
*N*	269	539	215	246	441	196
Female, *n* (%)	150 (55.8)	301 (55.8)	70 (32.6)	138 (56.1)	254 (57.6)	61 (31.1)
Race, *n* (%)						
Black	136 (50.6)	188 (34.9)	90 (41.9)	124 (50.4)	156 (35.4)	86 (43.9)
White	133 (49.4)	351 (65.1)	125 (58.1)	122 (49.6)	285 (64.6)	110 (56.1)
Age, mean (SD), years	39.8 (3.7)	40.5 (3.4)	40.9 (3.4)	44.9 (3.7)	45.5 (6.3)	45.7 (3.5)
IEAA, mean (SD), years	0.1 (4.4)	0.0 (4.3)	0.0 (4.2)	0.4 (4.1)	− 0.1 (4.5)	− 0.1 (4.4)
EEAA, mean (SD), years	− 0.4 (5.1)	− 0.1 (5.1)	0.7 (5.5)	− 0.2 (4.7)	− 0.1 (5.3)	− 0.1 (4.9)
PAA, mean (SD), years	− 0.3 (5.9)	0.1 (6.1)	0.2 (6.1)	0.0 (6.2)	− 0.2 (6.1)	0.3 (6.3)
GAA, mean (SD), years	− 1.3 (3.9)	− 0.3 (4.4)	2.4 (4.8)	− 1.2 (3.9)	− 0.3 (4.3)	2.0 (4.8)
Education, mean (SD), years	15.3 (2.4)	15.3 (2.6)	14.2 (2.4)	15.3 (2.4)	15.2 (2.6)	14.3 (2.4)
Center, *n* (%)						
Birmingham, AL	111 (41.3)	103 (19.1)	37 (17.2)	89 (36.1)	84 (19.0)	36 (18.4)
Chicago, IL	59 (21.9)	127 (23.6)	36 (16.7)	54 (22.0)	110 (24.9)	30 (15.3)
Minneapolis, MN	47 (17.5)	149 (27.6)	78 (36.3)	49 (19.9)	114 (25.9)	69 (35.2)
Oakland, CA	52 (19.3)	160 (29.7)	64 (29.8)	54 (22.0)	133 (30.2)	61 (31.1)
Tobacco smoking status, *n* (%)						
Never	231 (85.9)	340 (63.1)	73 (34.0)	218 (88.6)	250 (56.7)	72 (36.7)
Former	17 (6.3)	106 (19.7)	51 (23.7)	12 (4.9)	107 (24.3)	52 (26.6)
Current	21 (7.8)	93 (17.2)	91 (42.3)	16 (6.5)	84 (19.0)	72 (36.7)
Lifetime cigarette packs, mean (SD), packs	499.3 (1886.6)	1471.6 (2992.5)	3074.3 (3845.8)	403.6 (1568.1)	1843.5 (3285.5)	3109.4 (4148.0)
Physical activity, mean (SD), intensity score	297.1 (259.9)	351.3 (272.2)	405.4 (286.3)	293.8 (253.7)	349.8 (264.1)	413.1 (314.6)
BMI, mean (SD), kg/m^2^	29.4 (7.0)	28.2 (6.0)	28.6 (5.7)	30.3 (7.5)	29.0 (6.3)	29.0 (5.4)
Aspirin use ≥ 3 times per week, *n* (%)	12 (4.5)	35 (6.5)	13 (6.0)	30 (12.2)	54 (12.2)	25 (12.8)
Alcohol consumption, mean (SD), mL/day	4.3 (8.7)	11.1 (18.2)	24.2 (36.2)	4.6 (10.3)	11.6 (16.7)	24.4 (49.2)
Marijuana use in last 30 days, mean (SD), days	0 (0)	0.1 (0.4)	7.1 (9.6)	0 (0)	0.1 (0.5)	5.8 (9.2)

BMI, body mass index; IEAA, intrinsic epigenetic age acceleration; EEAA, extrinsic epigenetic age acceleration; PAA, PhenoAge acceleration; GAA, GrimAge acceleration; MJ, marijuana

### Cumulative marijuana use on epigenetic age acceleration

Table 2 presents the results for the association between cumulative marijuana use and EAA. After adjusting for covariates, ever using marijuana was positively associated with GAA at Y15 (*P* = 0.007). Ever use of marijuana was associated with a 0.71-year [95% CI 0.20, 1.23] higher GAA at Y15. Cumulative marijuana use was positively associated with GAA at Y15 (*P* < 0.001) and Y20 (*P* < 0.001) after adjusting for covariates. Specifically, there was a 0.25-year [95% CI 0.15, 0.36] and a 0.19-year [95% CI 0.11, 0.28] higher GAA per marijuana-year at Y15 and Y20, respectively. Results from GEE analyses yielded similar findings and conclusions as Y15 and Y20. We observed correlations, although weak, between marijuana-years and several GrimAge surrogate biomarkers of blood plasma proteins, including DNAm leptin, DNAm growth differentiation factor 15 (GDF15), DNAm cystatin C, and DNAm plasminogen activation inhibitor 1 (PAI1) (Additional file 1: Figure S1). IEAA, EEAA, and PAA were not associated with either ever use or cumulative marijuana use and results were unchanged after adjusting for aspirin use (data not shown).

**Table 2:** Analysis results for the association between cumulative marijuana use and EAA at examination years 15 and 20

	Year 15		Year 20		GEE	
	*β* [95% CI]	*P*	*β* [95% CI]	*P*	*β* [95% CI]	*P*
Ever marijuana use						
IEAA	− 0.07 [− 0.71, 0.57]	0.832	− 0.40 [− 1.10, 0.30]	0.264	− 0.24 [− 0.78, 0.30]	0.387
EEAA	0.26 [− 0.50, 1.02]	0.504	0.03 [− 0.76, 0.81]	0.950	0.14 [− 0.45, 0.73]	0.643
PAA	0.44 [− 0.45, 1.33]	0.336	− 0.39 [− 1.35, 0.58]	0.433	0.07 [− 0.63, 0.76]	0.852
GAA	0.71 [0.20, 1.23]	0.007	0.22 [− 0.33, 0.76]	0.430	0.49 [0.07, 0.90]	0.022
Cumulative marijuana use						
IEAA	− 0.04 [− 0.17, 0.09]	0.535	− 0.03 [− 0.15, 0.08]	0.558	− 0.04 [− 0.13, 0.05]	0.427
EEAA	0.04 [− 0.11, 0.20]	0.572	− 0.07 [− 0.20, 0.05]	0.237	− 0.03 [− 0.14, 0.08]	0.556
PAA	− 0.05 [− 0.24, 0.13]	0.564	− 0.02 [− 0.17, 0.14]	0.837	− 0.04 [− 0.18, 0.10]	0.578
GAA	0.25 [0.15, 0.36]	< 0.001	0.19 [0.11, 0.28]	< 0.001	0.21 [0.12, 0.30]	< 0.001

Results are adjusted for sex, race, center, education, tobacco smoking status, cumulative packs of cigarettes, BMI, physical activity, and alcohol consumption, with cumulative marijuana analyses further adjusted for ever marijuana use

Beta coefficient for ever marijuana use represents gain in EAA for ever users and beta coefficient for cumulative marijuana use represents gain in EAA for each additional marijuana-year

**Fig. 1:**
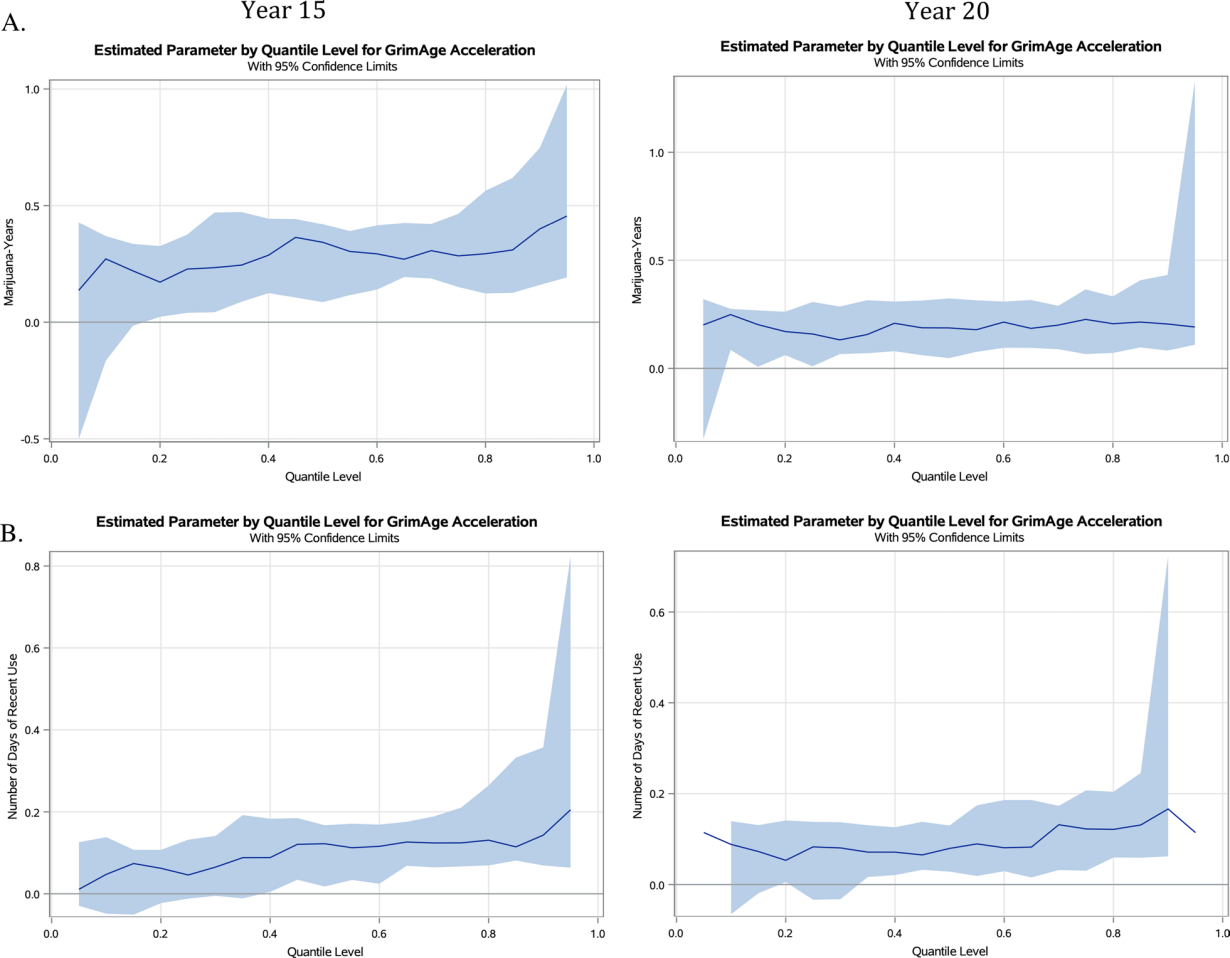
Estimated parameters by quantile with 95% confidence limits for the effect of cumulative and recent marijuana use on GrimAge acceleration at examination years 15 and 20. Quantile regression plots at Y15 and Y20 for **A** cumulative and **B** recent use of marijuana. The *x*-axis represents the quantile scale, and the *y*-axis represents the effect of marijuana use on GAA for a given quantile. Results are adjusted for sex, race, center, education, tobacco smoking status, cumulative packs of cigarettes, BMI, physical activity, and alcohol consumption, with cumulative marijuana analyses further adjusted for ever marijuana use

We further performed quantile regression to examine the effect of marijuana-years on GAA. Figure 1A presents plots from the quantile regression analyses for marijuana-years at Y15 and Y20 on GAA. Regression estimates were plotted for 19 quantiles ranging from 0.05 to 0.95. As displayed in the plots, the overall pattern depicts that marijuana-years has a positive association on GAA at both Y15 and Y20. The effect estimate of marijuana-years appears to be moderately flat at both Y15 and Y20, with a 0.25-year and a 0.19-year higher GAA for nearly all quantiles, respectively. These graphs demonstrate linear associations between marijuana-years and GAA.

### Recent marijuana use on epigenetic age acceleration

Table 3 presents the results for the association between recent marijuana use and EAA. Recent marijuana use was positively associated with GAA at Y15 (*P* < 0.001) and Y20 (*P* < 0.001). Compared to study participants who did not report using marijuana in the last 30 days, those who did had a 1.82-year [95% CI 1.16, 2.48] and 1.50-year [95% CI 0.78, 2.21] higher Y15 and Y20 GAA, respectively. The number of days of marijuana use in the last 30 days was also positively associated with GAA at both Y15 and Y20. Specifically, there was a 0.10-year [95% CI 0.05, 0.14] and a 0.10-year [95% CI 0.06, 0.15] higher GAA per day of marijuana use at Y15 and Y20, respectively. GEE results provided comparable associations at both Y15 and Y20. We observed similar weak correlations as marijuana-years between days of recent use and the GrimAge surrogate biomarkers of blood plasma proteins (Additional file 1: Figure S1). IEAA, EEAA, and PAA were not associated with either recent use or the number of days of recent use and results were unchanged after adjusting for aspirin use (data not shown).

**Table 3:** Analysis results for the association between recent marijuana use and EAA at examination years 15 and 20

	Year 15		Year 20		GEE	
	*β* [95% CI]	*P*	*β* [95% CI]	*P*	*β* [95% CI]	*P*
Recent marijuana use						
IEAA	− 0.42 [− 1.24, 0.41]	0.322	− 0.40 [− 1.32, 0.53]	0.402	− 0.39 [− 1.05, 0.28]	0.253
EEAA	0.39 [− 0.59, 1.37]	0.438	0.10 [− 0.93, 1.13]	0.852	0.26 [− 0.56, 1.07]	0.536
PAA	0.23 [− 0.92, 1.37]	0.699	0.30 [− 0.98, 1.58]	0.642	0.28 [− 0.74, 1.29]	0.593
GAA	1.82 [1.16, 2.48]	< 0.001	1.50 [0.78, 2.21]	< 0.001	1.70 [1.04, 2.37]	< 0.001
Recent marijuana use quantity						
IEAA	− 0.05 [− 0.11, 0.00]	0.050	− 0.02 [− 0.09, 0.04]	0.446	− 0.04 [− 0.08, 0.01]	0.097
EEAA	− 0.01 [− 0.07, 0.05]	0.752	− 0.02 [− 0.09, 0.05]	0.607	− 0.01 [− 0.07, 0.04]	0.667
PAA	− 0.04 [− 0.11, 0.04]	0.343	0.01 [− 0.08, 0.09]	0.865	− 0.02 [− 0.08, 0.05]	0.605
GAA	0.10 [0.05, 0.14]	< 0.001	0.10 [0.06, 0.15]	< 0.001	0.10 [0.06, 0.14]	< 0.001

Results are adjusted for sex, race, center, education, tobacco smoking status, cumulative packs of cigarettes, BMI, physical activity, and alcohol consumption

Beta coefficient for recent marijuana use represents gain in EAA for use in the last 30 days and beta coefficient for recent marijuana use quantity represents gain in EAA for each additional day within the last 30 days

Figure 1B presents plots from the quantile regression analyses for days of recent use at Y15 and Y20 on GAA. For Y15, the effect estimates of days of recent use gradually increased across the marijuana-GAA distribution, where the effect of days of recent use on GAA can be 3 times greater in the upper tail compared to the lower tail (i.e., 0.21-year vs 0.07-year higher GAA, respectively). A similar gradual increase was observed at Y20, with an approximately 3 times greater effect of days of recent use in the upper tail compared to the lower tail of the distribution (i.e., 0.16-year vs 0.05-year higher GAA, respectively).

### Marijuana use and alcohol consumption interaction on GrimAge acceleration

Table 4 presents the interaction and stratified analysis results for the joint association of marijuana use and alcohol consumption on GAA. At Y15, we observed a 0.22-year [95% CI 0.02, 0.42] higher GAA among nondrinkers compared to a 0.10-year [95% CI − 0.12, 0.31] higher GAA among heavy drinkers per marijuana-year (*P*_interaction_ = 0.017). Recent marijuana use was associated with a 1.56-year [95% CI 0.16, 2.97] higher GAA among nondrinkers compared to a 0.55-year [95% CI − 0.89, 1.98] higher GAA among heavy drinkers (*P*_interaction_ = 0.034). We also observed a 0.13-year [95% CI 0.05, 0.22] higher GAA among nondrinkers compared to a 0.04-year [95% CI − 0.05, 0.12] higher GAA for each additional day of recent use (*P*_interaction_ = 0.026).

**Table 4:** Interaction and stratified analysis results for the association between marijuana use and GAA at examination years 15 and 20 by strata of alcohol consumption

	Year 15		Year 20		GEE	
	*Β*_marijuana_ [95% CI]	*P*	*Β*_marijuana_ [95% CI]	*P*	*Β*_marijuana_ [95% CI]	*P*
Ever marijuana use	− **0.01 [**− **0.04, 0.02]**	**0.678**	− **0.04 [**− **0.07, 0.00]**	0.040*	− **0.02 [**− **0.06, 0.02]**	**0.386**
Nondrinker	0.96 [0.26, 1.66]	0.007	0.18 [− 0.59, 0.94]	0.645	0.58 [0.03, 1.13]	0.040
Light drinker	0.58 [− 0.61, 1.76]	0.338	0.15 [− 1.05, 1.35]	0.805	0.39 [− 0.29, 1.08]	0.260
Moderate drinker	0.30 [− 0.91, 1.51]	0.627	0.34 [− 0.93, 1.61]	0.596	0.37 [− 0.58, 1.31]	0.449
Heavy drinker	0.03 [− 2.06, 2.11]	0.981	− 1.69 [− 4.02, 0.64]	0.154	− 0.52 [− 2.53, 1.48]	0.609
Cumulative marijuana use	− **0.01 [**− **0.01, 0.00]**	0.017*	**0.00 [**− **0.01, 0.00]**	0.007*	− **0.01 [**− **0.01, 0.00]**	0.011*
Nondrinker	0.22 [0.02, 0.42]	0.035	0.22 [0.06, 0.37]	0.006	0.21 [0.05, 0.36]	0.008
Light drinker	0.58 [0.34, 0.81]	< 0.001	0.22 [− 0.13, 0.56]	0.221	0.48 [0.24, 0.72]	< 0.001
Moderate drinker	0.16 [− 0.04, 0.37]	0.115	0.35 [0.17, 0.53]	< 0.001	0.30 [0.15, 0.45]	< 0.001
Heavy drinker	0.10 [− 0.12, 0.31]	0.388	0.02 [− 0.14, 0.18]	0.776	0.05 [− 0.09, 0.18]	0.500
Recent marijuana use	− **0.02 [**− **0.04, 0.00]**	0.034*	− **0.03 [**− **0.05, **− **0.02]**	0.001*	− **0.03 [**− **0.04, **− **0.01]**	0.002*
Nondrinker	1.56 [0.16, 2.97]	0.029	1.30 [− 0.02, 2.61]	0.053	1.44 [0.09, 2.78]	0.036
Light drinker	3.51 [1.96, 5.05]	< 0.001	1.34 [− 0.87, 3.55]	0.233	2.93 [1.31, 4.54]	< 0.001
Moderate drinker	1.07 [− 0.12, 2.27]	0.079	1.93 [0.66, 3.19]	0.003	1.72 [0.72, 2.71]	< 0.001
Heavy drinker	0.55 [− 0.89, 1.98]	0.451	0.44 [− 1.27, 2.15]	0.610	0.61 [− 0.47, 1.68]	0.270
Recent marijuana use quantity	**0.00 [**− **0.01, 0.00]**	0.026*	**0.00 [0.00, 0.00]**	0.032*	**0.00 [0.00, 0.00]**	0.013*
Nondrinker	0.13 [0.05, 0.22]	0.003	0.12 [0.04, 0.21]	0.006	0.13 [0.05, 0.22]	0.002
Light drinker	0.23 [0.13, 0.34]	< 0.001	0.06 [− 0.10, 0.22]	0.475	0.18 [0.07, 0.29]	0.001
Moderate drinker	0.01 [− 0.07, 0.09]	0.748	0.16 [0.06, 0.26]	0.001	0.09 [0.00, 0.17]	0.045
Heavy drinker	0.04 [− 0.05, 0.12]	0.366	0.02 [− 0.07, 0.11]	0.648	0.03 [− 0.03, 0.10]	0.343

*Interaction terms with *P* ≤ 0.05

Bolded values represent the beta coefficient [95% CI] and *P* for the joint association between marijuana use and alcohol consumption

Results are adjusted for sex, race, center, education, tobacco smoking status, cumulative packs of cigarettes, BMI, and physical activity, with cumulative marijuana analyses further adjusted for ever marijuana use

Beta coefficient for ever marijuana use, cumulative marijuana use, recent marijuana use, and recent marijuana use quantity represents gain in GAA for ever users, for each additional marijuana-year, use in the last 30 days, and for each additional day within the last 30 days, respectively

Compared to the Y15 interaction analysis results, similar but more significant associations were observed at Y20. We observed a 0.22-year [95% CI 0.06, 0.37] higher GAA among nondrinkers compared to a 0.02-year [95% CI − 0.14, 0.18] loss in GAA among heavy drinkers per marijuana-year (*P*_interaction_ = 0.007). For recent marijuana use, we observed a 1.30-year [95% CI − 0.02, 2.61] higher GAA among nondrinkers compared to a 0.44-year [95% CI − 1.27, 2.15] higher GAA among heavy drinkers (*P*_interaction_ = 0.001). For the number of days of recent marijuana use, we observed a 0.12-year [95% CI 0.04, 0.21] and a 0.02-year [95% CI − 0.07, 0.11] higher GAA per day among nondrinkers and heavy drinkers, respectively (*P*_interaction_ = 0.032). Interaction and stratified results from GEE provided similar findings. While interactions of the marijuana variables with tobacco smoking status, race, and sex on GAA yielded primarily non-significant associations, former smokers, White participants, and male participants displayed higher GAA with marijuana use compared to never and current smokers, Black participants, and female participants, respectively (Additional file 1: Tables S1–S3).

## Discussion

We observed positive associations between cumulative and recent marijuana use and GAA in young adults. We observed ever use of marijuana and each additional marijuana-year were associated with a 6-month and 2.5-month higher GAA average, respectively. Additionally, any recent use, which exhibited the largest effect estimate, and each additional day of recent use were associated with a 20-month and 1-month higher GAA average, respectively. We also observed statistical interactions between cumulative and recent marijuana use and alcohol consumption on GAA, with nondrinkers exhibiting a higher average in GAA compared to heavy drinkers. These findings provide novel insights into the association between marijuana use and epigenetic age acceleration as estimated by GAA.

As a DNA-methylation-based measure of biological age, GrimAge is a composite biomarker of seven DNA methylation surrogates. Several of these surrogates of GAA have been associated with components of the endocannabinoid system, including leptin [31], GDF15 [32], cystatin C [33], and PAI1 [34]. We observed similar, albeit weak, correlations between several GrimAge surrogate biomarkers of blood plasma proteins and marijuana in our study, suggesting the association between marijuana and GAA may occur through DNA methylation changes related to these specific plasma proteins. When comparing correlations between the GrimAge surrogate biomarkers of blood plasma proteins and marijuana use and cumulative packs of cigarettes, we note despite only a modest correlation between these variables (*r* = 0.11–0.25), their correlations with surrogate biomarkers were generally consistent in direction but smaller in magnitude for marijuana use. This suggests marijuana and tobacco use may operate via similar pathways. The associations between marijuana and GAA remained robust even after adjustment for cumulative packs of cigarettes, suggesting epigenetic age-related changes are independent of cigarette smoking. Additionally, the observed variation in associations between the four EAA metrics may be due to the methodological differences in the development of these measures, which capture different aspects of the aging process. Together, the current and previous results demonstrate marijuana may modulate DNA methylation-based surrogate biomarkers associated with lifespan and may negatively impact the aging process. Given the movement to legalize marijuana, interventions to limit marijuana use may aid in slowing the aging process and potentially, hinder age-related conditions and improve longevity. However, further studies examining marijuana and its effect on GAA and corresponding blood plasma proteins may provide new mechanistic insight into the molecular effects of this health-related behavior and its effects on disease risk.

The magnitude of effect of marijuana on age-related epigenetic changes appear to differ by the period of exposure to marijuana. Although recent use of marijuana exhibited a three times greater gain in GAA compared to ever use of marijuana during GEE analysis, marijuana-years exhibited a greater gain in GAA compared to the number of days of recent use, suggesting the large effect of recent exposure is also transient (at least with regards to its effect on GAA). This may reflect the pharmacokinetics of cannabis where plasma concentrations of metabolites, such as tetrahydrocannabinol, are highest after use and decrease over time [35]. The higher concentration and rapid decline in blood tetrahydrocannabinol concentration with recent use may result in temporary epigenetic alterations that subsequently resolve over time. However, prolonged use may lead to the accumulation of marijuana metabolites in adipose tissue that are released into the blood and subsequently, exert sustained effects on blood DNA methylation [35, 36]. As such, behavioral modifications to limit use of marijuana may aid in limiting both short- and long-term impacts on the aging process as captured through DNA methylation.

Marijuana is the most commonly used controlled substance among those who consume alcohol [37]. We observed cumulative and recent marijuana use were associated with a higher GAA among nondrinkers compared to drinkers, who exhibited a smaller GAA gain with increasing alcohol intake. While these findings suggest statistical interactions between marijuana and alcohol, the biological mechanism for this interaction remains unclear. Alcohol consumption has previously been shown to increase cytokine production and subsequent peripheral inflammation and damage to organs [38, 39], and cannabis may exert anti-inflammatory properties and mitigate inflammation from alcohol consumption [40–42], suggesting opposing effects of cannabis and alcohol on inflammatory pathways. Inflammatory marker IL-6 was previously found to be positively associated with alcohol consumption and further analysis identified a statistical interaction between alcohol consumption and marijuana use, where a significant positive association was observed among non-users and a non-significant negative association was observed among users, demonstrating marijuana may modulate inflammatory cytokines induced by alcohol [43]. Studies have also observed cannabinoids may reduce alcohol-induced oxidative stress and autophagy related damage [40, 44]. In sum, our statistical findings are consistent with proposed mechanisms and findings demonstrating opposing effects of marijuana use in the context of alcohol consumption. Additional studies are needed to explore the relationship between marijuana use, alcohol consumption, and inflammation, as well as potential lifestyle modifications to mitigate molecular/epigenetic damage and long-term health risks.

The large study sample and longitudinal nature of CARDIA allowed us to obtain repeated methylation levels and marijuana data, enabling us to explore the association of marijuana on the aging process at multiple time points. Furthermore, as a US cohort, CARDIA enables for a better assessment of the independent effects of marijuana and tobacco on health outcomes due to the lower frequency of marijuana mixed with tobacco compared to other countries [45]. This study is not without limitations. Marijuana use among study participants may have been underreported due to social desirability bias. However, the questionnaire was self-administered, given at a research facility, and responses were confidential [23]. Furthermore, underreporting would likely skew our results towards the null and thus, associations presented here are likely underestimates of the true associations. Additionally, the observed associations may be due to how marijuana was used, i.e., smoked, where inhaled intoxicants may also contribute to age-related epigenetic changes, compared to other forms of use (e.g., vaporized, edible, etc.). Lastly, due to different measures of EAA and marijuana at different time points, this study inherently yielded multiple analyses. Correction for multiple testing was not performed due to analyses being primarily non-independent and to avoid hindering future investigations [46].

## Conclusions

In conclusion, we observed significant associations between cumulative and recent marijuana use and GrimAge acceleration. We also found statistical interactions between marijuana and alcohol on GAA. Our findings provide novel insights into the potential association of marijuana use on the aging process as captured by blood DNA methylation age-related changes. Epigenetic aging provides a unique approach to elucidate epigenetic age-related changes and has the potential to serve as biomarker for disease development and potentially lifespan. Given the growing aging population and the increasing trend of legalization in the USA, understanding the effects of marijuana on the epigenome may provide novel information and its effect on the aging process.

## Supplementary Information

**Additional file 1:** **Supplemental Table 1.** Interaction and stratified analysis results for the association between marijuana use and GrimAge acceleration at examination years 15 and 20 by tobacco smoking status. **Supplemental Table 2.** Interaction and stratified analysis results for the association between marijuana use and GrimAge acceleration at examination years 15 and 20 by race. **Supplemental Table 3.** Interaction and stratified analysis results for the association between marijuana use and GrimAge acceleration at examination years 15 and 20 by sex. **Supplemental Figure 1.** Pairwise correlation of marijuana use, cumulative packs of cigarettes, and DNA methylation-based biomarkers of GrimAge at examination years 15 and 20.

## Data Availability

The epigenetic datasets generated and analyzed are available from the corresponding author upon reasonable request.

## Abbreviations

BMI: Body mass index
CARDIA: Coronary Artery Risk Development in Young Adults
EAA: Epigenetic age acceleration
EEAA: Extrinsic epigenetic age acceleration
IEAA: Intrinsic epigenetic age acceleration
GAA: GrimAge acceleration
GEE: Generalized estimating equation
PAA: PhenoAge acceleration
