# Supplementary material for: Marijuana use and DNA methylation-based biological age in young adults
Source: Clin Epigenetics. 2022 Oct 26;14:134. doi: 10.1186/s13148-022-01359-8 (PMC9609285; doi:10.1186/s13148-022-01359-8)
Supplement: Supplementary file 1 — Additional file 1: Supplemental Table 1. Interaction and stratified analysis results for the association between marijuana use and GrimAge acceleration at examination years 15 and 20 by tobacco smoking status. Supplemental Table 2. Interaction and stratified analysis results for the association between marijuana use and GrimAge acceleration at examination years 15 and 20 by race. Supplemental Table 3. Interaction and stratified analysis results for the association between marijuana use and GrimAge acceleration at examination years 15 and 20 by sex. Supplemental Figure 1. Pairwise correlation of marijuana use, cumulative packs of cigarettes, and DNA methylation-based biomarkers of GrimAge at examination years 15 and 20. [file 13148_2022_1359_MOESM1_ESM.docx]

**Supplemental Table 1.** Interaction and stratified analysis results for the association between marijuana use and GrimAge acceleration at examination years 15 and 20 by tobacco smoking status

|  | Year 15 | |  | Year 20 | |  | GEE | |
| --- | --- | --- | --- | --- | --- | --- | --- | --- |
|  | Β_marijuana_ [95% CI] | *P* |  | Β_marijuana_ [95% CI] | *P* |  | Β_marijuana_ [95% CI] | *P* |
| Ever Marijuana Use | **0.01 [-0.72, 0.73]** | **0.989** |  | **-0.06 [-0.86, 0.74]** | **0.883** |  | **-0.03 [-0.80, 0.74]** | **0.934** |
| Never Smoker | 0.65 [0.10, 1.19] | 0.021 |  | 0.01 [-0.57, 0.58] | 0.980 |  | 0.37 [-0.06, 0.80] | 0.091 |
| Former Smoker | 0.73 [-1.13, 2.59] | 0.439 |  | 0.53 [-1.42, 2.49] | 0.591 |  | 0.49 [-1.20, 2.18] | 0.567 |
| Current Smoker | 0.67 [-1.06, 2.40] | 0.446 |  | 0.03 [-1.96, 2.02] | 0.977 |  | 0.29 [-1.36, 1.93] | 0.734 |
|  |  |  |  |  |  |  |  |  |
| Cumulative Marijuana Use | **-0.10 [-0.21, 0.02]** | **0.093** |  | **-0.07 [-0.17, 0.02]** | **0.139** |  | **-0.08 [-0.17, 0.02]** | **0.117** |
| Never Smoker | 0.30 [0.13, 0.47] | <0.001 |  | 0.20 [0.06, 0.33] | 0.004 |  | 0.23 [0.08, 0.38] | 0.002 |
| Former Smoker | 0.47 [0.25, 0.70] | <0.001 |  | 0.34 [0.18, 0.49] | <0.001 |  | 0.38 [0.25, 0.51] | <0.001 |
| Current Smoker | 0.11 [-0.08, 0.31] | 0.253 |  | 0.10 [-0.08, 0.29] | 0.272 |  | 0.11 [-0.04, 0.26] | 0.147 |
|  |  |  |  |  |  |  |  |  |
| Recent Marijuana Use | **0.27 [-0.44, 0.98]** | **0.458** |  | **-0.67 [-1.45, 0.10]** | **0.090** |  | **-0.12 [-0.79, 0.56]** | **0.735** |
| Never Smoker | 1.36 [0.38, 2.33] | 0.007 |  | 1.83 [0.81, 2.85] | <0.001 |  | 1.55 [0.52, 2.58] | 0.003 |
| Former Smoker | 2.29 [0.76, 3.82] | 0.004 |  | 2.59 [1.15, 4.03] | <0.001 |  | 2.42 [1.01, 3.84] | <0.001 |
| Current Smoker | 1.96 [0.72, 3.19] | 0.002 |  | 0.45 [-1.09, 2.00] | 0.564 |  | 1.51 [0.48, 2.53] | 0.004 |
|  |  |  |  |  |  |  |  |  |
| Recent Marijuana Use Quantity | **-0.03 [-0.08, 0.01]** | **0.151** |  | **-0.01 [-0.06, 0.05]** | **0.777** |  | **-0.02 [-0.07, 0.02]** | **0.348** |
| Never Smoker | 0.10 [0.03, 0.18] | 0.004 |  | 0.08 [0.01, 0.14] | 0.021 |  | 0.09 [0.02, 0.16] | 0.013 |
| Former Smoker | 0.22 [0.11, 0.33] | <0.001 |  | 0.19 [0.10, 0.28] | <0.001 |  | 0.20 [0.11, 0.28] | <0.001 |
| Current Smoker | 0.05 [-0.02, 0.12] | 0.194 |  | 0.10 [-0.01, 0.20] | 0.078 |  | 0.07 [0.01, 0.13] | 0.032 |

Bolded values represent the beta coefficient [95% CI] and *P* for the joint association between marijuana use and tobacco smoking status.

Results are adjusted for sex, race, center, education, cumulative packs of cigarettes, BMI, physical activity, and alcohol consumption, with cumulative marijuana analyses further adjusted for ever marijuana use.

**Supplemental Table 2.** Interaction and stratified analysis results for the association between marijuana use and GrimAge acceleration at examination years 15 and 20 by race

|  | Year 15 | |  | Year 20 | |  | GEE | |
| --- | --- | --- | --- | --- | --- | --- | --- | --- |
|  | Β_marijuana_ [95% CI] | *P* |  | Β_marijuana_ [95% CI] | *P* |  | Β_marijuana_ [95% CI] | *P* |
| Ever Marijuana Use | **0.84 [-0.12, 1.80]** | **0.087** |  | **0.79 [-0.23, 1.80]** | **0.128** |  | **0.77 [-0.00, 1.55]** | **0.051** |
| Black Participants | 0.31 [-0.50, 1.13] | 0.450 |  | -0.12 [-0.99, 0.76] | 0.795 |  | 0.16 [-0.51, 0.82] | 0.647 |
| White Participants | 1.12 [0.44, 1.80] | 0.001 |  | 0.49 [-0.22, 1.20] | 0.178 |  | 0.81 [0.31, 1.31] | 0.002 |
|  |  |  |  |  |  |  |  |  |
| Cumulative Marijuana Use | **0.27 [0.07, 0.46]** | **0.009^*^** |  | **0.07 [-0.09, 0.23]** | **0.404** |  | **0.15 [-0.02, 0.32]** | **0.082** |
| Black Participants | 0.09 [-0.10, 0.28] | 0.349 |  | 0.16 [0.02, 0.31] | 0.027 |  | 0.14 [0.00, 0.28] | 0.058 |
| White Participants | 0.36 [0.23, 0.48] | <0.001 |  | 0.20 [0.09, 0.30] | <0.001 |  | 0.26 [0.14, 0.38] | <0.001 |
|  |  |  |  |  |  |  |  |  |
| Recent Marijuana Use | **0.71 [-0.53, 1.95]** | **0.262** |  | **0.67 [-0.68, 2.02]** | **0.331** |  | **0.68 [-0.52, 1.88]** | **0.264** |
| Black Participants | 1.49 [0.42, 2.55] | 0.006 |  | 1.18 [0.04, 2.33] | 0.043 |  | 1.42 [0.56, 2.29] | 0.001 |
| White Participants | 2.19 [1.33, 3.04] | <0.001 |  | 1.68 [0.75, 2.62] | <0.001 |  | 1.94 [0.98, 2.91] | <0.001 |
|  |  |  |  |  |  |  |  |  |
| Recent Marijuana Use Quantity | **0.12 [0.04, 0.20]** | **0.004^*^** |  | **0.01 [-0.08, 0.10]** | **0.778** |  | **0.07 [-0.01, 0.15]** | **0.078** |
| Black Participants | 0.03 [-0.04, 0.10] | 0.344 |  | 0.10 [0.02, 0.17] | 0.014 |  | 0.06 [0.00, 0.13] | 0.061 |
| White Participants | 0.16 [0.10, 0.21] | <0.001 |  | 0.10 [0.04, 0.16] | 0.001 |  | 0.13 [0.07, 0.19] | <0.001 |

^*^ Interaction terms with *P* ≤ 0.05.

Bolded values represent the beta coefficient [95% CI] and *P* for the joint association between marijuana use and race.

Results are adjusted for sex, center, education, tobacco smoking status, cumulative packs of cigarettes, BMI, physical activity, and alcohol consumption, with cumulative marijuana analyses further adjusted for ever marijuana use.

**Supplemental Table 3.** Interaction and stratified analysis results for the association between marijuana use and GrimAge acceleration at examination years 15 and 20 by sex

|  | Year 15 | |  | Year 20 | |  | GEE | |
| --- | --- | --- | --- | --- | --- | --- | --- | --- |
|  | Β_marijuana_ [95% CI] | *P* |  | Β_marijuana_ [95% CI] | *P* |  | Β_marijuana_ [95% CI] | *P* |
| Ever Marijuana Use | **-0.32 [-1.27, 0.63]** | **0.510** |  | **-0.33 [-1.32, 0.67]** | **0.521** |  | **-0.31 [-1.08, 0.46]** | **0.432** |
| Male Participants | 1.05 [0.23, 1.86] | 0.012 |  | 0.30 [-0.52, 1.13] | 0.472 |  | 0.70 [0.08, 1.32] | 0.026 |
| Female Participants | 0.37 [-0.28, 1.02] | 0.259 |  | -0.02 [-0.75, 0.71] | 0.957 |  | 0.19 [-0.36, 0.75] | 0.498 |
|  |  |  |  |  |  |  |  |  |
| Cumulative Marijuana Use | **0.01 [-0.21, 0.23]** | **0.943** |  | **-0.02 [-0.20, 0.17]** | **0.855** |  | **0.00 [-0.18, 0.18]** | **0.964** |
| Male Participants | 0.28 [0.15, 0.41] | <0.001 |  | 0.20 [0.09, 0.30] | <0.001 |  | 0.22 [0.11, 0.33] | <0.001 |
| Female Participants | 0.21 [0.02, 0.40] | 0.034 |  | 0.13 [-0.04, 0.30] | 0.125 |  | 0.16 [0.01, 0.32] | 0.044 |
|  |  |  |  |  |  |  |  |  |
| Recent Marijuana Use | **-0.66 [-1.92, 0.60]** | **0.303** |  | **-1.82 [-3.21, -0.42]** | **0.011^*^** |  | **-1.18 [-2.34, -0.01]** | **0.048^*^** |
| Male Participants | 2.33 [1.40, 3.26] | <0.001 |  | 2.16 [1.21, 3.10] | <0.001 |  | 2.30 [1.37, 3.23] | <0.001 |
| Female Participants | 1.11 [0.15, 2.07] | 0.023 |  | 0.18 [-0.92, 1.29] | 0.743 |  | 0.70 [-0.11, 1.51] | 0.092 |
|  |  |  |  |  |  |  |  |  |
| Recent Marijuana Use Quantity | **-0.05 [-0.14, 0.04]** | **0.270** |  | **-0.01 [-0.11, 0.09]** | **0.857** |  | **-0.03 [-0.12, 0.06]** | **0.506** |
| Male Participants | 0.12 [0.07, 0.18] | <0.001 |  | 0.11 [0.05, 0.16] | <0.001 |  | 0.11 [0.06, 0.17] | <0.001 |
| Female Participants | 0.03 [-0.03, 0.10] | 0.323 |  | 0.07 [-0.01, 0.16] | 0.101 |  | 0.06 [-0.02, 0.13] | 0.127 |

^*^ Interaction terms with *P* ≤ 0.05.

Bolded values represent the beta coefficient [95% CI] and *P* for the joint association between marijuana use and sex.

Results are adjusted for race, center, education, tobacco smoking status, cumulative packs of cigarettes, BMI, physical activity, and alcohol consumption, with cumulative marijuana analyses further adjusted for ever marijuana use.

**Supplemental Figure 1.** Pairwise correlation of marijuana use, cumulative packs of cigarettes, and DNA methylation-based biomarkers of GrimAge at examination years 15 and 20


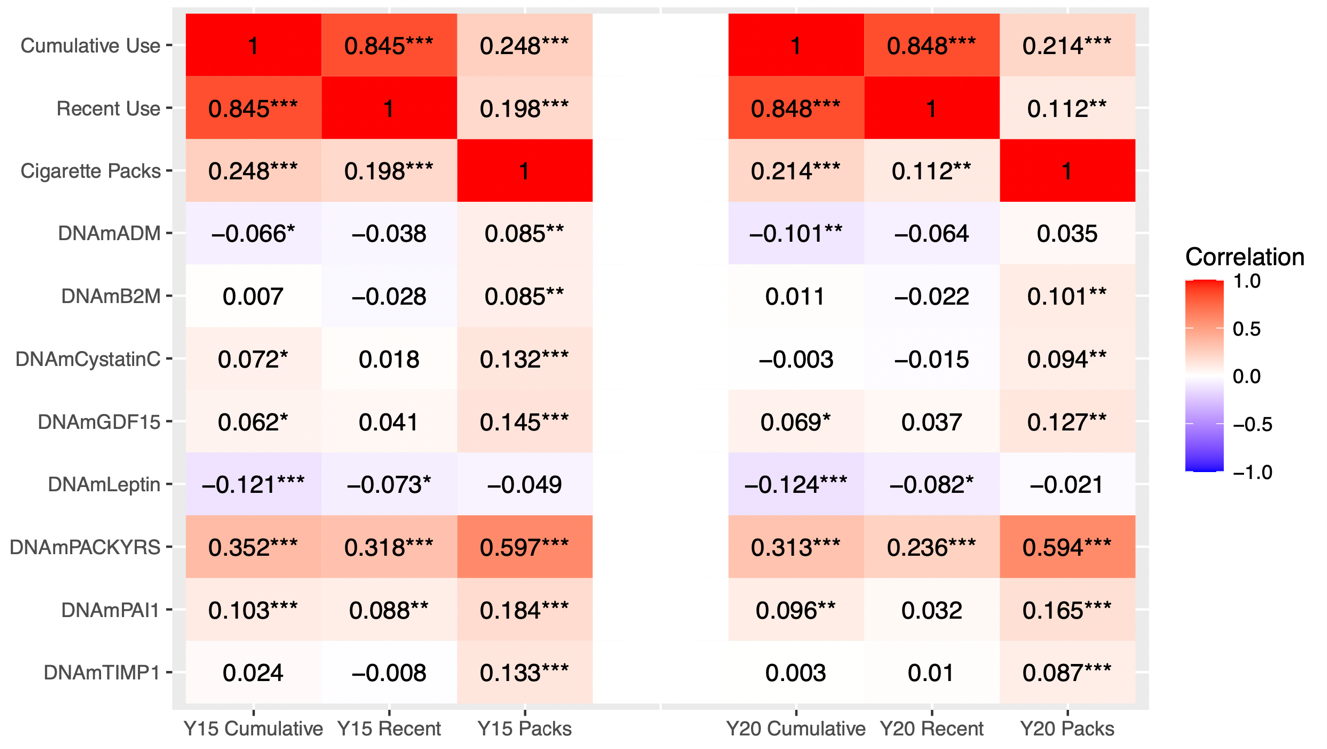


The columns represent marijuana-years, number of days of recent use, and cumulative packs of cigarettes for Y15 and Y20 and the rows represent the DNA methylation-based biomarkers of GrimAge with the Pearson correlation coefficient reported within each box, respectively. ****P*<0.001, ***P*<0.01, **P*<0.05.

DNAmADM, adrenomedullin; DNAmB2M, beta-2 microglobulin; DNAmCystatinC, cystatin C; DNAmGDF15, growth differentiation factor 15; DNAmLeptin, leptin; DNAmPACKYRS, smoking pack-years; DNAmPAI1, plasminogen activation inhibitor 1; DNAmTIMP1, tissue inhibitor metalloproteinase 1.
